# FELASA Congress 2025

**DOI:** 10.1002/ame2.70133

**Published:** 2025-12-30

**Authors:** Lai Wei

**Affiliations:** ^1^ The Chinese Association for Laboratory Animal Sciences Beijing China

**Keywords:** animal welfare and ethics, FELASA, laboratory animal sciences

## Abstract

The FELASA 2025 Congress, themed “Reducing Severity in Animal Research,” convened global experts in laboratory animal sciences to discuss ethical advancements, welfare standards, and innovative practices. Key sessions highlighted the integration of the 3R principles (Replacement, Reduction, Refinement), with presentations addressing challenges in immunology, AI‐driven animal monitoring, and aseptic surgical techniques. Notable lectures, including Prof. Stasinos Stavrianeas' exploration of zoology's origins and Dr. David Anderson's award‐winning talk on squid welfare, emphasized cultural relevance and ethical responsibility. The congress also prioritized sustainability through waste‐reduction measures and digital tools like an official app for enhanced networking. Exhibitions showcased cutting‐edge technologies, while workshops fostered hands‐on skill development. By advocating openness and interdisciplinary collaboration, FELASA 2025 aimed to advance humane and scientifically rigorous animal research practices globally.

## Graphical abstract

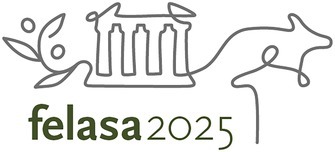

## 1 FELASA 2025

Themed “Reducing Severity in Animal Research,” FELASA 2025 Congress attracted experts and scholars from the global laboratory animal sciences field. The event featured a diverse range of sessions, including keynote speeches, themed workshops, 180‐s oral presentations, commercial seminars, and ePosters. The academic presentations delved into several key areas, such as demonstrating ethical awareness and responsibility, reducing severity, enhancing care and welfare standards, fostering a culture of compassion, pursuing a better future, and specialized sessions on skill enhancement techniques. These discussions covered topics such as promoting accurate statistical reporting on the creation and breeding of genetically modified animals in the EU, focusing on surgical training by providing tools for those involved in laboratory animal surgery training and assessment, conducting hazard–benefit assessments when using complex mouse models in neuroscience experiments, exploring AI‐driven advanced animal monitoring and birth detection technologies, sharing the latest training methods and practical experiences for mice and rats, understanding the principles and practical methods of aseptic surgery in rodents, and addressing strategies for trainees to handle special difficulties during practical training. The congress also advocated for the application of animal welfare and the “3R” principles (replacement, reduction, refinement), using lectures and workshops to examine the current landscape and envision the future of laboratory animal science research.

## 2 FELASA SESSIONS

### 2.1 Presentations

There are three keynote lectures in 2025 FELASA Congress. Prof. Stasinos Stavrianeas gave a lecture on “The Origins of Zoology and Aristotle's Biology.” Prof. George Paxinos talked about “Is the Brain in the Goldilocks Zone?”. Dr. David Anderson, who won the FELASA Award this year, talked about his experience “From Calving Cows to Championing Squid Welfare.”

It is noteworthy that Prof. Stasinos Stavrianeas is not the only one who related lecture to local culture. Wendy Jarrett shared the attitudes toward animals shown by great thinkers and how they may have shaped our modern ethical thinking. These lectures intertwined with local characteristics demonstrate the scholars’ respect to the local culture and may foster emotional bond between participants and the city where the congress is held. Thereby, it can be a great way to enhance international collaboration. The diversified cultural characteristics can enrich the congress's content with new perspectives, creating a vibrant atmosphere to stimulate new idea.

Focusing on the topic of “Reducing Severity in Animal Research,” many scientists chose 3R to be the theme of their presentations. Prof. Santiago F. Gonzalez delivered a presentation titled “Challenges of Applying 3Rs in the Field of Immunology.” The speech began with the explanation of why the application of 3R can be difficult. For example, in some inflammatory disease models, pain and distress caused to animals are inevitable. Then solutions were proposed to address these difficulties. Among them, the 4R, which represents the reuse of scientific data, is a promising one. The IMMUNEMAP was introduced as a platform that supports data‐driven immunological research by sharing data from research laboratories around the world.

AI has also been a popular topic in the congress. In the session of Al‐Driven Advanced Animal Monitoring and Birth Detection: Enhancing Animal Welfare and Research Precision, Prof. Jan‐Bas Prins introduced “Birth detection in mice: an innovative acoustic approach (DVC2.0).” The acoustic signals can be collected by microphones in cages. Then, they can be analyzed using AI, which can identify specific vocal patterns. In this way, the births of pups can be detected automatically with precision. The modification of cages and the application of new technologies not only meet the needs of scientific research but also contribute to enhancing the welfare of laboratory animals. Recently, *AMEM* has also published an article focused on improving husbandry for Lepr^db/db^ mouse.^1^ The new protocol proposed in this article has led to fewer complications. In addition to filling the gap in research, AI can be handy to administrative work. Dr. Filipe Nunes shared his experience applying AI in his daily work in the presentation called “AI to the Rescue: How AI Tools Can Help NACWOs, NTCOs, and NIOs Roles.” The use of Microsoft Copilot can ease the workload for administrators working in laboratory animal sciences. However, he also warned against being dependent on AI tools and suggested responsible training, use, and oversight.

As last FELASA Congress's motto, communication still takes a great part in this year's FELASA Congress. There were several presentations on communication. This year's discussion on communication has included both employees from the field of laboratory animal sciences and the public. “Openness” was a point that has been mentioned multiple times. In the presentation “Why Openness Works” by Dr. Stanley and Dr. Addelman, a majority of the public in the UK feels not very well informed about animal research, whereas more than half consider using animals for research acceptable as long as there is no alternative or unnecessary suffering to the animals. It means that better demonstration of scientists’ effort fighting for better medical sciences can raise acceptance and understanding of those who work with laboratory animals. Eventually, the animals themselves can be the beneficiary. The Concordat on Openness on Animal Research was introduced in Hannah Hobson's presentation. The bodies signing the concordat are committed to disclose their status of using animals in research. The public can make informed decision based on the latest information in the concordat annual report. In addition to introducing the concordat, she shared some advice on how to talk about harms. One of them is that it should be admitted that research and testing can bring suffering to animals. This is also stressed by Mary Harvie in her presentation on “Empowering Scientists and Techs to Talk Confidently about Animal Research on Social Media.” She also warned against neglecting animals receiving harm in research. Furthermore, she suggested that people be realistic without using the actual images of animals’ wounds. She also emphasized that we should keep in mind that this is a serious subject, and animals’ sacrifice made for science should not be trivialized. She also gave inspiring notes on conversations and dealing with negativity, which is very common on social media. Perhaps the most valuable take‐home information is that even though negativity is unavoidable in many conversations, abuse should not be accepted, and blocking, deleting people, or even hiding can be helpful.

### 2.2 Exhibition

An exhibition showcased the latest technologies and products in laboratory animal infrastructure, environmental control systems, and experimental instruments, highlighting the deep integration of academic research and technological innovation. The congress received support from five gold sponsors, one silver sponsor, and three bronze sponsors, with an additional 84 organizations participating as exhibitors. Besides manufacturers of experimental instruments and equipment, training institutions took part in the exhibition.

## 3 HIGHLIGHTS IN FELASA 2025

### 3.1 Humanistic care

#### 3.1.1 Environmental friendly congress

This FELASA put waste reduction at its core. The brochure of agenda and basic congress information was small and easy to carry. Apart from that, there was no other material or congress bag. It may seem inconvenient, but this assumption was far from the truth. Beside the registration desk, the titles, times, and locations of the various programs were displayed on a board. In the congress hall, the signs leading to different conference rooms were quite clear. The cloakroom was open during the congress, so there was no need to worry about carrying heavy stuff while attending the congress. The employees in red T‐shirts were always ready to help. With these sufficient preparation, there was no confusion or inconvenience for attendants. In the meanwhile, the potential waste and pollution of producing brochures and bags were successfully avoided.

#### 3.1.2 Equality

Hygiene products and first‐aid packages were provided at the registration desk. The notes were stuck on the mirrors in the ladies’ rooms. To avoid embarrassment, a code was set so that attendants could receive the products discreetly. This thoughtful gesture can greatly enhance participants’ experience and boost satisfaction. The culture of care was not only discussed in talks but also demonstrated through practical actions.

### 3.2 Registration and booking

The FELASA 2025 included many kinds of workshops giving chances of group work and hands‐on experience. However, additional booking was required for all the workshops. It was a process that was completed before the start of the congress. In this case, almost all places of workshops were booked. If an attendee wanted to join any workshop without a reservation, then the attendee should wait to see if any place could be open due to someone who reserved a place not turning up. However, high passion continued throughout the whole congress. In a word, booking in advance is essential to a fruitful experience.

### 3.3 The official APP and website

The FELASA 2025 has launched an official APP for all participants. It includes floor plans, agenda, legends of icons, and explanations of abbreviations. During the congress, the APP would send words from sponsors to users as promotion for sponsors. Some other notifications were also sent from time to time. This app was featured with functions of messaging people and highlighting interested programs. The featured functions were very helpful in replacing printed materials.

### 3.4 Networking

Another key character of FELASA is the abundant opportunities to network. The time span for lunch break and tea time was long enough to move from small talks to serious conversations. Besides, there were only smaller, higher tables in the venue, so all participants had to stay closer to each other. What's more, the organizers also held activities specifically for networking such as congress evening and charity run. These activities were inclusive enough to allow participants to have more chances to communicate with each other in a vibrant atmosphere.

## FUNDING INFORMATION

The author has nothing to report.

## CONFLICT OF INTEREST STATEMENT

Lai Wei is an editor of Animal Models and Experimental Medicine (*AMEM*) and the author of this article. To minimize bias, she was excluded from all editorial decision making related to the acceptance of this article for publication.

## ETHICS STATEMENT

Not applicable.
